# Information Processing in the Brain as Optimal Entropy Transport: A Theoretical Approach

**DOI:** 10.3390/e22111231

**Published:** 2020-10-29

**Authors:** Carlos Islas, Pablo Padilla, Marco Antonio Prado

**Affiliations:** 1 Universidad Autónoma de la Ciudad de México, Doctor García Diego núm. 168, Cuauhtémoc, Ciudad de México 06720, Mexico; carlos.islas@uacm.edu.mx (C.I.); marcoantonio.prado@uacm.edu.mx (M.A.P.); 2 IIMAS, Universidad Nacional Autónoma de México Circuito Escolar, Cd. Universitaria, Ciudad de México 04510, Mexico

**Keywords:** informational entropy, neuroscience, Monge–Ampère equation, optimal transport, variational calculus, Murray’s law, neuronal branching structures

## Abstract

We consider brain activity from an information theoretic perspective. We analyze the information processing in the brain, considering the optimality of Shannon entropy transport using the Monge–Kantorovich framework. It is proposed that some of these processes satisfy an optimal transport of informational entropy condition. This optimality condition allows us to derive an equation of the Monge–Ampère type for the information flow that accounts for the branching structure of neurons via the linearization of this equation. Based on this fact, we discuss a version of Murray’s law in this context.

## 1. Introduction

The brain as the organ responsible for processing information in the body has been subjected to evolutionary pressure. “The business of the brain is computation, and the task it faces is monumental. It must take sensory inputs from the external world, translate this information into a computationally accessible form, then use the results to make decisions about which course of action is appropriate given the most probable state of the world” [1]. It is therefore arguable that information processing has been optimized, at least to some extent, by natural selection [2,3]. This is a rather abstract claim that should ultimately be contrasted with experiments (in this respect, see [4,5]). In a subsequent work, we explore this question in more detail, but a plausible connection can be established with experimental and theoretical results via fMRI in which different measures of cost have been proposed. The brain as an informational system is the subject of active research (see for instance [6,7,8]). For example, “behavior analysis has adopted these tools [Information theory] as a novel means of measuring the interrelations between behavior, stimuli, and contingent outcomes” [1]. In this context, in [1], it was shown that informational measures of contingency appear to be a reasonable basis for predicting behavior. We also refer the reader to the issue devoted to information theory in neuroscience [9] for a recent account of this perspective. In the work just mentioned, several papers investigate optimization principles (for instance, the maximum entropy principle [10] or the free energy principle; see [11,12]) as tools for understanding inference, coding, and other brain functionalities. Information theory will allow filling the gaps between disciplines, for example psychology, neurobiology, physics, mathematics, and computer science. In the present paper, we adopt a related setting and mathematically formalize information processing in the brain within the framework of optimal transport. The rationale for this is consistent with the view that some essential brain functionalities such as inference and the coordination of tasks (e.g., auditory and motor activities) involve the transportation of information and that such processes should be efficient, have been subjected to evolutionary pressure, and as a consequence, are (pseudo) optimal. As was already pointed out, our theoretical proposal should be contrasted with experimental results.

It is necessary to observe from the very beginning that information processing and transport are of an intrinsically spatiotemporal nature. Therefore, our proposal should include these two features. In doing so, we expect the spatial part of the optimization to give rise to spatial patterns, for instance network-like or branching hierarchical components, as well as the temporal structure, such as periodic or synchronized patterns. However, in this paper, we begin by considering only the spatial part, except for a few general remarks. Since our proposal is an attempt to establish a methodological framework to study informational entropy transport in the brain, we deal with spacial aspects first, leaving a study of the dynamical aspects for a subsequent work. Furthermore, in order to simplify the problem, we consider only the one-dimensional case in the mathematical formalism. However, it is certain that the geometry of the brain has to play an essential role in all the processes [13], and we extrapolate some of the results we obtain to two and three dimensions.

Now, we provide an overview of the paper. In Section 2, we present a general framework for information processing in the brain as an optimal transport of entropy, as well as some mathematical results in the context of the Monge–Kantorovich problem. The main idea is that instead of considering that some sort of physical mass is being transported, it is informational entropy. In order to provide the mathematical results and for the sake of completeness, we adapt the material on the existence of a solution in the optimal mass transportation case as presented by Bonnotte [14] to the informational case. We conclude this section with a derivation of the Monge–Ampère equation in the one-dimensional case. We begin Section 3 by recalling the linearization of the Monge–Ampère equation around the square of the distance function, which involves the Laplacian. Then, we argue that adding a nonlinear term is justified by the physiological nature of transmission along neurons. The resulting model is a semilinear elliptic equation. At the end of this section, we relate the qualitative features of the solutions with the branching structure of neural networks in the brain. In other words, we show that the optimal transport process of informational entropy is consistent with the geometric branching structure of neural branching. In Section 4, we elaborate on the relationship of the branching structure of neurons and Murray’s law [15], which provides the optimal branching ratio of the father to the daughter branch sections, as well as the optimal bifurcation angle. We propose a modified version of Murray’s law when the underlying transport network carries information instead of a fluid. The last section is devoted to concluding remarks, further research, and open questions.

## 2. The Monge–Kantorovich Problem

We present a general overview of the results on optimal transportation theory needed in this work. For a complete exposition, see [14,16,17] or [18], but for the sake of completeness, we include a general discussion without giving the proofs of the results, but including appropriate references. Our presentation follows closely [14] and some parts of [16].

The original Monge–Kantorovich problem was formulated in the context of mass transport (Monge) or budget allocation (Kantorovich). In a later section, we will adapt the setting to include the transport of informational entropy.

Monge’s problem: Given two probability measures μ and ν on $R^{n}$ and a cost function $c:R^{n}\times R^{n}⟶\left[0,\infty \right]$, the problem of Monge can be stated as follows:

(1) $$\mathrm{Find}\;T:R^{n}⟶R^{n},\;\mathrm{such}\;\mathrm{that}\;\nu =T\#\mu \;\mathrm{and}\int_{R^{n}}c\left(x,T\left(x\right)\right)d\mu \left(x\right)\;\mathrm{is}\;\mathrm{minimal}.$$

The condition ν=T#μ means that *T* transports μ onto ν; that is, ν is the push-forward of μ by *T*: for any ξ, $\int_{R^{n}}\xi \left(y\right)d\nu \left(y\right)=\int_{R^{n}}\xi \left(T\left(x\right)\right)d\mu \left(x\right)$.

Monge–Kantorovich problem: Monge’s problem might have no solution; hence, it is better to take the following generalization proposed by Leonid Kantorovich: instead of looking for a map, find a measure:

(2) $$\pi \in \Pi \left(\mu ,\nu \right)\;\mathrm{such}\;\mathrm{that}\;\int_{R^{n}\times R^{n}}c\left(x,y\right)d\pi \left(x,y\right)\;\mathrm{is}\;\mathrm{minimal},$$

where Π(μ,ν) stands for the set of all transport plans between μ and ν, i.e., the probability measures on $R^{n}\times R^{n}$ with marginals μ and ν. This problem really extends Monge’s problem. For any transport map *T*, sending μ onto ν yields a measure π∈Π(μ,ν), which is given by π=(Id,T)#μ, i.e., the only measure π on $R^{n}\times R^{n}$ such that:

$$\forall \xi \in C_{b},\int_{R^{n}\times R^{n}}\xi \left(x,y\right)d\pi \left(x,y\right)=\int_{R^{n}}\xi \left(T\left(x\right)\right)d\mu \left(x\right),$$

and the associated costs of transportation are the same. In this version, it is not difficult to show that there is always a solution ([14] or [16]).

### 2.1. Dual Formulation

There is a duality between the Monge–Kantorovich problem (2) and the following problem:

$$\mathrm{Find}\;\psi \in L^{1}\left(\mu \right),\phi \in L^{1}\left(\nu \right),\;\mathrm{such}\;\mathrm{that}\;\psi \left(x\right)+\phi \left(y\right)\leq c\left(x,y\right)\;\mathrm{and}\;\int_{X}\psi \left(x\right)d\mu +\int_{Y}\phi \left(y\right)d\nu \;\mathrm{is}\;\mathrm{maximal}.$$

It seems natural to look for a solution of this problem among the pairs (ψ,ϕ)∈P(X×Y) that satisfy:

$$\begin{array}{c} \phi \left(y\right)=\underset{x}{\inf}\left\{c\left(x,y\right)-\psi \left(x\right)\right\} \end{array}\;\mathrm{and}\;\begin{array}{c} \psi \left(x\right)=\underset{y}{\inf}\left\{c\left(x,y\right)-\phi \left(y\right)\right\}. \end{array}$$

We will write $\phi \left(y\right)=\psi^{c}\left(y\right)$ and $\psi \left(x\right)=\phi^{c}\left(x\right)$.

**Definition** **1.** *A function ψ is said to be c-concave if $\psi =\phi^{c}$ for some function ϕ. In that case, $\psi^{c}$ and $\phi^{c}$ are called the c-transform of ψ and ϕ, respectively. We also say that $\left(\psi ,\psi^{c}\right)$ is an admissible pair and ψ, $\psi^{c}$ are admissible potentials.*

Then, the problem becomes:

(3) $$\mathrm{Find}\;\psi \in L^{1}\left(\mu \right)\;\mathrm{such}\;\mathrm{that}\;\int_{X}\psi \left(x\right)d\mu +\int_{Y}\psi^{c}\left(y\right)d\nu \;\mathrm{is}\;\mathrm{maximal}.$$

The function ψ is called a Kantorovich potential between μ and ν.

The following proposition explains how to relate the Monge–Kantorovich problem (2) with (3), known as the Kantorovich duality principle.

**Proposition** **1** (Kantorovich duality principle)**.** *Let μ and ν be Borel probability measures on X and $Y\in R^{n}$, respectively. If the cost function $c:R^{n}\times R^{n}⟶\left[0,\infty \right]$ is lower semi-continuous and:*

$$\int_{Y}\int_{X}c\left(x,y\right)d\mu \left(x\right)d\nu \left(y\right)<\infty ,$$

*then there is a Borel map $\psi :R^{n}⟶R$ that is c-concave and optimal for *(3)*. Moreover, the resulting maximum is equal to the minimum of the Monge–Kantorovich problem *(2)*; i.e.,*

$$\min_{\pi \in \Pi (\mu ,\nu )}\int_{R^{n}\times R^{n}}c\left(x,y\right)d\pi \left(x,y\right)=\max_{\phi \in L^{1}\left(\mu \right)}\left\{\int_{X}\phi \left(x\right)d\mu \left(x\right)+\int_{Y}\phi^{c}\left(y\right)d\nu \left(y\right)\right\},$$

*or:*

(4) $$\min_{\pi \in \Pi (\mu ,\nu )}I\left[\pi \right]=\max_{(\phi ,\phi^{c})\in \Phi }J\left(\phi ,\phi^{c}\right),$$

*where,*

(5) $$\Phi =\left\{\left(\phi ,\psi \right)\in L^{1}\left(\mu \right)\times L^{1}\left(\nu \right)|\phi \left(x\right)+\psi \left(y\right)\leq c\left(x,y\right)\;\;\mathrm{for}\;\mu -a.e.\;x\in X\;\mathrm{and}\;\nu -a.e.\;y\in Y\right\}.$$

*If π∈Π(μ,ν) is optimal, then $\phi \left(x\right)+\phi^{c}\left(y\right)=c\left(x,y\right)$ almost everywhere for π.*

**Proof.**  A proof of this result can be found in [16]. □

### 2.2. Solution in the Real Line: Optimal Transportation Case

For the rest of this section, we only consider the one-dimensional case, as discussed in the Introduction.

Let *X* and *Y* be two bounded smooth open sets in R and μ(dx), ν(dy) the probability measures of *X* and *Y*, respectively, with μ(dx)=fdx, ν(dy)=gdy, f=0 in R\*X*, and g=0 in R\*Y*. Let F:R⟶[0,1] and G:R⟶[0,1] be the cumulative distributions of μ and ν, respectively, defined by F(x)=μ((−∞,x]) and G(y)=ν((−∞,y]).

**Proposition** **2.** *Let h *∈*$C^{1}\left(R\right)$ be a non-negative, strictly convex function. Let μ and ν be Borel probability measures on R such that:*

(6) $$\int_{Y}\int_{X}h\left(x-y\right)d\mu \left(x\right)d\nu \left(y\right)<\infty .$$

*If μ has no atom and F and G stand for the respective cumulative distribution of μ and ν, respectively, then:*

$$T=G^{-1}\circ F$$

*solves Monge’s problem *(1)* for the cost:*

(7) c(x,y)=h(x−y).

*If π is the induced transform plan, that is:*

π=(Id,T)#μ

*defined as $T\#\mu \left(E\right)=\mu (T^{-1}\left(E\right))$ for E∈Y, then π is optimal for the Monge–Kantorovich problem *(2)*.*

**Proof.**  A proof of Proposition (2) can be found in [14]. □

In order to get the previous result, one has to consider the functional:

$$\begin{array}{c} \int_{X\times Y}c\left(x,y\right)d\pi \left(x,y\right), \end{array}$$

where c:X×Y⟶R is some given cost function and Π(x,y) stands for the set of all transport plans between μ and ν, meaning the probability measures on R×R with marginals μ and ν; i.e.,

(8) $$\Pi \left(\mu ,\nu \right)=\left\{\pi \in P\left(X\times Y\right)|\int_{Y}d\pi \left(x,y\right)=d\mu \left(x\right),\int_{X}d\pi \left(x,y\right)=d\nu \left(y\right)\right\},$$

or more rigorously:

$$\Pi \left(\mu ,\nu \right)=\left\{\pi \in P(X\times Y)|\pi (A\times Y)=\mu (A),\pi (X,B)=\nu (B),A\subseteq X,B\subseteq Y\right\}.$$

Our goal is to get a similar result to Proposition (2) in the case when entropy transportation is considered instead of mass transportation. This is the content of the next section.

## 3. Solution in the Real Line: Optimal Entropy Transportation Case

As was pointed out in [1], “the fundamental measure in information theory is the entropy. It corresponds to the uncertainty associated with a signal. “Entropy” and “information” are used interchangeably, because the uncertainty about an outcome before it is observed, corresponds to the information gained by observing it” (for a review, see [1,19] or [20]). In that context, we will prove the existence of an optimal entropy transport for the cost function c(x,y) satisfying (6) and (7), similarly to the optimal transportation case discussed in the last section. We will also find the Monge–Ampère equation for quadratic cost ${\left|x-y\right|}^{2}/2$ for this optimal entropy transport. A few words are in order regarding the choice of *c*. From the mathematical perspective, it considerably simplifies the analysis. As a matter of fact, the optimal transportation problem has not been solved for the general case of nonquadratic costs. On the other hand, from the physiological point of view, it is natural to assume that the energy required to send a signal from one point of the brain to another can be taken as a monotone function of the distance.

Let μ and ν be probability measures defined as above, then take X=Y=Ω∈R, and let the entropy be characterized by Shannon’s proposal:

(9) −ρ(x)ln(ρ(x)),

where x∈Ω, ρ, and $\tilde{\rho }$ are the distribution densities with respect to μ and ν, respectively, in the same way as the formulation of the optimal transportation problem states; i.e.,

$$\mu =\rho dx,\;\nu =\tilde{\rho }dy,$$

satisfying (8). We wish (9) to be related to the probability measure μ in Ω. It is natural to think that in passing from the state characterized by (9) to the one characterized by $-\tilde{\rho }\left(y\right)\ln\left(\tilde{\rho }\left(y\right)\right)$, where y∈Ω and wish it to be related to the probability measure ν in Ω. Similarly, −ρ(x)ln(ρ(x)) and $-\tilde{\rho }\left(y\right)\ln\left(\tilde{\rho }\left(y\right)\right)$ will be the marginals of −ρ(x,y)ln(ρ(x,y)).

As a first concrete proposal, we consider the following functional:

$$\int_{\Omega \times \Omega }c\left(x,y\right)\rho \left(x,y\right)\ln\left(\rho \left(x,y\right)\right)\;dxdy,$$

where *c* is a spatial cost function. Notice that we have dropped the minus sign, so looking for maximal entropy is equivalent to minimizing the previous expression. The problem is then to find the optimal entropy transport strategy between *x* and *y* (analogous to Monge’s problem). There is however a standard problem, which is the fact that in the continuous case, the entropy could be negative, whereas in the discrete case (i.e., for discrete probability functions), the entropy is always positive. We therefore consider the absolute value of the entropy defined in (9). More precisely, define the following:

$$\left|\rho \ln\rho \left(x\right)\right|={\left(\rho \ln\rho \right)}^{+}\left(x\right)+{\left(\rho \ln\rho \right)}^{-}\left(x\right),$$

with:

$$\begin{array}{c} {\left(\rho \ln\rho \right)}^{+}\left(x\right)=\max_{\Omega }\left(\rho \ln\rho ,0\right) \end{array}\;\mathrm{and}\;\begin{array}{c} {\left(\rho \ln\rho \right)}^{-}\left(x\right)=-\min_{\Omega }\left(\rho \ln\rho ,0\right). \end{array}$$

It is natural to assume that:

(10) $$\int_{\Omega }\left|\rho \ln\rho \left(x\right)\right|\;dx=K,$$

for some constant $K\in R^{+}\backslash${0}, and let:

(11) $$d\mu =\frac{1}{K}\left|\rho \left(x\right)\ln\rho \left(x\right)\right|dx,$$

with:

$$\begin{array}{c} d\mu^{+}=\frac{1}{K}{\left(\rho \ln\rho \right)}^{+}\left(x\right)dx \end{array}\;\mathrm{and}\;\begin{array}{c} d\mu^{-}=\frac{1}{K}{\left(\rho \ln\rho \right)}^{-}\left(x\right)dx. \end{array}$$

**Observation** **1.** *If ρ(x,y) is the distribution density of π(x,y) in Ω×Ω, then *(11)* is a well-defined Borel probability measure on Ω, and:*

(12) $$d\nu =\frac{1}{K}\left|\tilde{\rho }\left(y\right)\ln\tilde{\rho }\left(y\right)\right|dy$$

*is also a well-defined Borel probability measure on Ω. Then, we can define:*

(13) $$\hat{\Pi }\left(\mu ,\nu \right)=\left\{\pi \in P\left(\Omega \times \Omega \right)|\int_{Y=\Omega }d\pi \left(x,y\right)=d\mu \left(x\right),\int_{X=\Omega }d\pi \left(x,y\right)=d\nu \left(y\right)\right\},$$

*with μ and ν given by *(11)* and *(12)*; or more rigorously:*

$$\begin{array}{c} \hat{\Pi }\left(\mu ,\nu \right)=\left\{\pi \in P(\Omega \times \Omega )|\pi (A,\Omega )=\mu (A),\pi (\Omega ,B)=\nu (B),A,B\subseteq \Omega \right\}, \end{array}$$

*with μ and ν given by *(11)* and *(12)*; we can take the cumulative distributions of μ and ν, respectively, by F(x)=μ((−∞,x]) and G(y)=ν((−∞,y]) with μ and ν given as before. By definition, they are non-decreasing.*

Now, we are in the condition to establish the problem (1) analogous to that of Monge, namely:

(14) $$\begin{array}{c} \mathrm{Find}\;a\;\mathrm{map}\;T:R⟶R,\;\;\mathrm{such}\;\mathrm{that}\;\nu =T\#\mu \;\;\mathrm{with}\;\;\;\mu \;\mathrm{and}\;\nu \;\;\mathrm{given}\;\mathrm{by}\;(11)\;\mathrm{and}\;(12)\;\mathrm{and}\\ \int_{\Omega }c\left(x,T\left(x\right)\right)d\mu \left(x\right)\;\mathrm{is}\;\mathrm{minimal},\;\;\;\;\;\;\; \end{array}$$

and analogous to the Monge–Kantorovich problem:

(15) $$\begin{array}{c} \mathrm{Find}\;a\;\mathrm{measure}\;\pi \in \hat{\Pi }\left(\mu ,\nu \right)\;\mathrm{given}\;\mathrm{by}\;\left(13\right)\;\mathrm{with}\;\mu \;\mathrm{and}\;\nu \;\mathrm{given}\;\mathrm{by}\;\left(11\right)\;\mathrm{and}\;\left(12\right)\;\mathrm{such}\;\mathrm{that}\\ \int_{\Omega \times \Omega }c\left(x,y\right)\;d\pi \left(x,y\right)\;\;\mathrm{is}\;\mathrm{minimal}.\;\;\;\;\;\;\; \end{array}$$

Next, we present the equivalent proposition of (2), for the measures given by (11) and (12), namely:

**Proposition** **3.** *Let h∈$C^{1}\left(R\right)$ be a non-negative, strictly convex function. Let μ and ν be Borel probability measures on R given by *(11)* and *(12)*, respectively. Suppose that:*

(16) $$\int_{\Omega }\int_{\Omega }h\left(x-y\right)d\mu \left(x\right)d\nu \left(y\right)<\infty .$$

*for Ω∈R. If μ has no atom and F and G represent the corresponding cumulative distribution functions of μ and ν, respectively, then:*

$$T=G^{-1}\circ F$$

*solves Problem *(14)* for the cost:*

c(x,y)=h(x−y).

*If π is the induced entropy transform plan, that is:*

π=(Id,T)#μ,

*defined as $T\#\mu \left(E\right)=\mu (T^{-1}\left(E\right))$ for E∈Ω, then π is optimal for Problem *(15)*.*

**Proof.**  The proof of this result is adapted from [14] and for the sake of completeness is given in Appendix A. □

Our next goal is to deduce the Monge–Ampère equation for this case. In order to do that, we will need an analog of the Kantorovich duality principle (Proposition (1)), for the measures given by (11) and (12), namely:

**Proposition** **4.** *Let μ and ν be the Borel probability measures on Ω∈R given by *(11)* and *(12)*, respectively. If the cost function:*

c:R×R⟶[0,∞)

*is lower semi-continuous and:*

$$\int_{\Omega }\int_{\Omega }c\left(x,y\right)d\mu \left(x\right)d\nu \left(y\right)<\infty ,$$

*then there is a Borel map ψ:R⟶R that is c-concave and optimal for *(3)*. Moreover, the resulting maximum is equal to the minimum of Problem *(15)*; i.e.,*

$$\min_{\pi \in \hat{\Pi }\left(\mu ,\nu \right)}\int_{R\times R}c\left(x,y\right)d\pi \left(x,y\right)=\max_{\psi \in L^{1}\left(\mu \right)}\left\{\int_{\Omega }\psi \left(x\right)d\mu \left(x\right)+\int_{\Omega }\psi^{c}\left(y\right)d\nu \left(y\right)\right\}$$

*or:*

(17) $$\min_{\pi \in \hat{\Pi }\left(\mu ,\nu \right)}I\left[\pi \right]=\max_{(\psi ,\psi^{c})\in \Phi }J\left(\psi ,\psi^{c}\right),$$

*where,*

(18) $$\Phi =\left\{\left(\phi ,\psi \right)\in L^{1}\left(\mu \right)\times L^{1}\left(\nu \right)\;|\;\phi \left(x\right)+\psi \left(y\right)\leq c\left(x,y\right)\;\mathrm{for}\;\mu -a.e.\;x\in \Omega \;\mathrm{and}\;\nu -a.e.\;y\in \Omega \right\},$$

*and if $\pi \in \hat{\Pi }\left(\mu ,\nu \right)$ given by *(13)* is optimal, then $\psi \left(x\right)+\psi^{c}\left(y\right)=c\left(x,y\right)$ almost everywhere for π.*

**Proof.**  A proof of this result can be found in Appendix A. □

If we propose $C\left(x,y\right)={\left|x-y\right|}^{2}/2$, we wish *T* to be expressed as T=∇ϕ for some convex function ϕ and then to be able to find the corresponding Monge–Ampère equation related to the measures μ and ν given by (11) and (12). This fact is guaranteed by Brenier’s theorem. The details of its proof adapted for our case are important and can be found in Appendix A.

**Theorem** **1** (Brenier)**.** *Let μ and ν be the Borel probability measures on Ω∈R given by *(11)* and *(12)*, respectively, and with finite second-order moments; that is, such that:*

(19) $$\begin{array}{c} \int_{\Omega }{\left|x\right|}^{2}d\mu \left(x\right)<\infty \end{array}\;\mathrm{and}\;\begin{array}{c} \int_{\Omega }{\left|y\right|}^{2}d\nu \left(y\right)<\infty . \end{array}$$

*Then, if μ is absolutely continuous on *Ω*, there exists a unique*

*T:R⟶R such that ν=T#μ and:*

$$\int_{\Omega }{\left|x-T\left(x\right)\right|}^{2}d\mu \left(x\right)=\min_{\gamma \in \hat{\Pi }\left(\mu ,\nu \right)}\int_{\Omega }{\left|x-y\right|}^{2}d\gamma \left(x,y\right),$$

*with $\hat{\Pi }\left(\mu ,\nu \right)$ given by *(13)*. Moreover, there is only one optimal transport plan, γ, which is necessarily (Id,T)#μ, and T is the gradient of a convex function φ, which is therefore unique up to an additive constant. There is also a unique (up to an additive constant) Kantorovich potential, ψ, which is locally Lipschitz and linked to φ through the relation:*

$$\varphi \left(x\right)=\frac{1}{2}{\left|x\right|}^{2}-\psi \left(x\right).$$

**Proof.**  See Appendix A. □

**Observation** **2.** *Observe that Theorem *1* holds for the general case on $R^{n}$.*

### The Monge–Ampère Equation

Let:

$$\begin{array}{c} d\mu \left(x\right)=\frac{1}{K}|\rho \left(x\right)\ln\left[\rho \left(x\right)\right]|dx \end{array}\;\mathrm{and}\;\begin{array}{c} d\nu \left(y\right)=\frac{1}{K}|\tilde{\rho }\left(y\right)\ln\left[\tilde{\rho }\left(y\right)\right]|dy \end{array}$$

be two probability measures, absolutely continuous with respect to the Lebesgue measure. By Theorem 1, there exists a unique gradient of a convex function, φ, such that:

(20) $$\int_{\Omega }\zeta \left(y\right)|\tilde{\rho }\left(y\right)\ln\left[\tilde{\rho }\left(y\right)\right]|dy=\int_{\Omega }\zeta \left(\nabla \phi \left(x\right)\right)|\rho \left(x\right)\ln\left[\rho \left(x\right)\right]|dx,$$

for all test functions $\zeta \in C_{b}\left(R\right)$. Since φ is strictly convex, then ∇φ is $C^{0}$ and one-to-one. Hence, taking y=∇φ(x), we get:

(21) $$\int_{\Omega }\zeta \left(y\right)|\tilde{\rho }\left(y\right)\ln\left[\tilde{\rho }\left(y\right)\right]|dy=\int_{\Omega }\zeta \left(\nabla \phi \left(x\right)\right)|\tilde{\rho }\left(\nabla \varphi \left(x\right)\right)\ln\left[\tilde{\rho }\left(\nabla \varphi \left(x\right)\right)\right]|\det D^{2}\varphi \left(x\right)dx.$$

From (20) and (21), we get:

(22) $$|\rho \left(x\right)\ln\left[\rho \left(x\right)\right]|=|\tilde{\rho }\left(\nabla \varphi \left(x\right)\right)\ln\left[\tilde{\rho }\left(\nabla \varphi \left(x\right)\right)\right]|\det D^{2}\varphi \left(x\right),$$

and the Monge–Ampère equation:

(23) $$\det D^{2}\varphi \left(x\right)=\frac{\left|\rho (x)\ln[\rho (x)]\right|}{\left|\tilde{\rho }\left(\nabla \varphi \left(x\right)\right)\ln\left[\tilde{\rho }\left(\nabla \varphi \left(x\right)\right)\right]\right|},$$

corresponding to this case.

## 4. Neural Branching Structure and the Linearization of the Monge–Ampère Equation

As was pointed out in the Introduction, the purpose of this section is to propose a model for the branching structure of the neurons, which is consistent with the process of information transport previously introduced. The basic idea is as follows. If we consider that information transport is optimized in some brain processes, we consequently have (as discussed in the previous section) an associated Monge–Ampère equation for the transport plan potential. Besides, it is natural to consider a cost that is close to some power of the distance function, since physiological cost can be taken to depend on the distance traveled by the corresponding signal, as is usually assumed in transport networks. For technical reasons, and in order to be able to adapt results well known in the literature, we take this cost function to be close to:

$$\frac{{\left|x-y\right|}^{2}}{2},$$

From a qualitative perspective, this choice should not change the results much, as long as the cost function remains convex (see [16]). If this is the case, we can then compute the linearization of the Monge–Ampère equation around this quadratic cost function. The resulting equation is a linear elliptic equation. We argue that a self-activating mechanism should be incorporated in the form of a nonlinear term (as a result of the excitable nature of the transport of electric impulses along axons). In this way, we end up with a semilinear equation that can be used to explain branching processes in biological networks (see [21]). More specifically, the solution to this equation could be associated with the concentration of a morphogen, e.g., a growth factor, and if such a concentration is above a certain threshold, a branching mechanism is triggered. It is then consistent to look for solutions that are close to the quadratic cost function and therefore to linearize around it.

In order to linearize the Monge–Ampère equation, we assume then that φ is very close to ${\left|x\right|}^{2}/2$, so |ρ(x)ln[ρ(x)]| is very close to $\left|\tilde{\rho }\left(\nabla \varphi \left(x\right)\right)\ln\left[\tilde{\rho }\left(\nabla \varphi \left(x\right)\right)\right]\right|$. In that case, following [16,22,23], make:

(24) $$\varphi \left(x\right)\equiv \varphi_{\varepsilon }\left(x\right)=\frac{{\left|x\right|}^{2}}{2}+\varepsilon \eta +O\left(\varepsilon^{2}\right)$$

and:

(25) $$|\tilde{\rho }\left(\nabla \varphi \left(x\right)\right)\ln\left[\tilde{\rho }\left(\nabla \varphi \left(x\right)\right)\right]|=|\tilde{\rho }\left(\nabla \varphi \left(x\right)\right)\ln\left[\tilde{\rho }\left(\nabla \varphi \left(x\right)\right)\right]{|}_{\varepsilon }\equiv \left(1+\varepsilon h+O\left(\varepsilon^{2}\right)\right)|\rho \left(x\right)\ln\left[\rho \left(x\right)\right]|,$$

with $\eta ,h\in L^{1}\left(\mu \right)$ and ε≪1. We leave the details of this computation for Appendix A. Substituting (24) and (25) in the Monge–Ampère Equation (23), we get as the linearized operator:

(26) Lη=h,

with:

$$L=\left(-\Delta +\nabla \left(-\log|\rho (x)\ln\rho (x)|\right)\cdot \nabla \right).$$

Then, the Laplacian plus a transport term can be seen as the linearized version of the Monge–Ampère equation for our proposal. We notice that the main mechanism responsible for the flow of information along the axons is the propagation of electrical impulses. This is well known to be an excitable process that involves, among others, a self-activating component, as for instance in the standard Hodgkin–Huxley or FitzHugh–Nagumo models. If we include this into the linearization previously obtained (Equation (26)), we get:

$$L\phi =\left(-\Delta \phi +\nabla \left(-\log|\rho (x)\ln\rho (x)|\right)\cdot \nabla \phi \right)+F\left(\phi \right),$$

where *F* a is function describing the self-activating mechanism and can be taken typically as a power of ϕ:

$$F\left(\phi \right)=\phi^{p},$$

with p>1. Solutions of this type of equations have been studied by many authors since the pioneering work by Ni and Takagi ([24] and the references therein), since they appear in different contexts. These solutions typically exhibit concentrations that can be responsible for branching structures.

**Figure 1:**
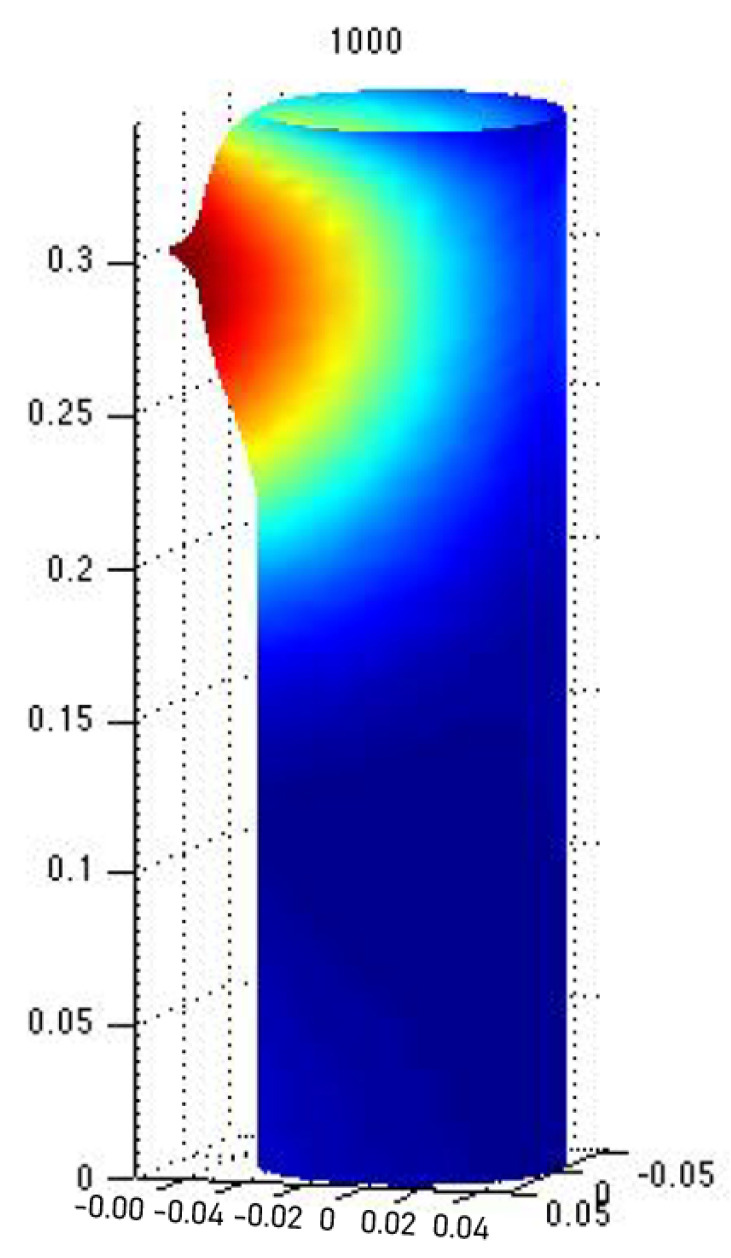
Numerical solution of the linearization of the Monge-Ampère equation including growth. Branching occurs when there is the concentration of the solution, the morphogen, above a certain threshold (the color code stands for standard heat maps: red, high; blue, low). This simulation was provided by Jorge Castillo-Medina and developed in COMSOL. For more details, the reader is referred to [21] and the references therein.

**Figure 2:**
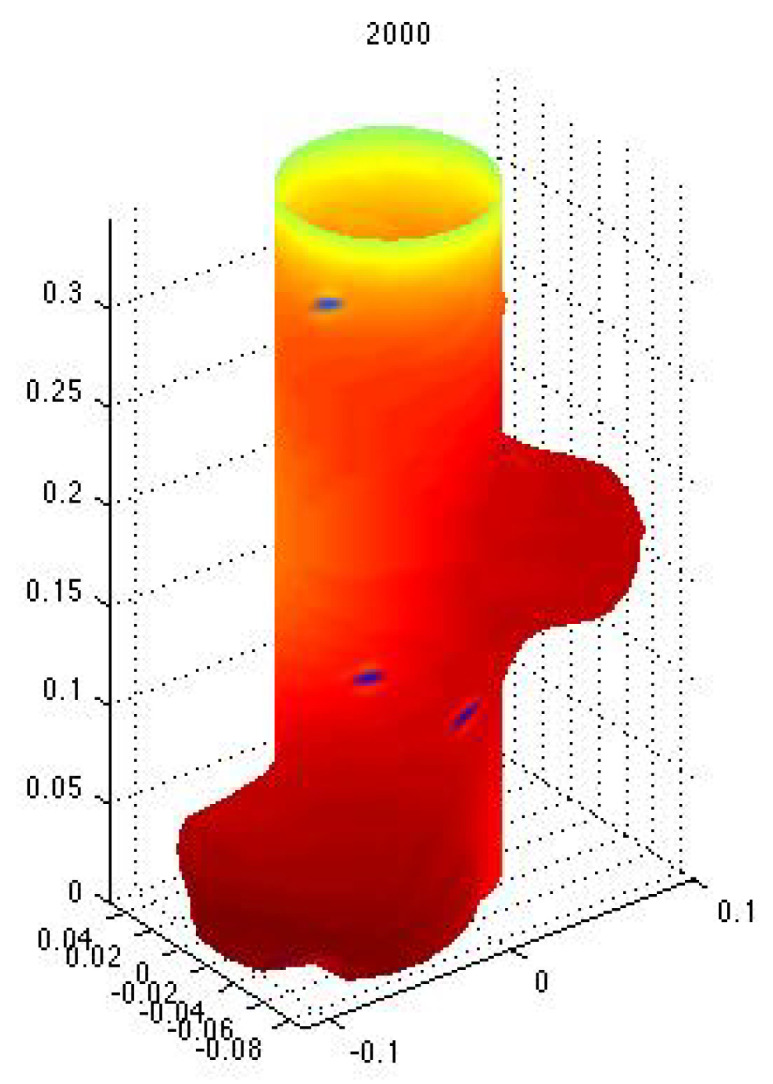
A similar simulation as in the previous figure with a different growth rate. Notice the different branching structure. Simulation performed by J. Castillo as well; see also [21].

Indeed, if one assumes that the concentration of a solution to the previous equation is correlated with a growth factor morphogen, then a branch will stem out of the main branch. This or similar models have been proposed using reaction-diffusion models following Turing’s original proposal ([25] or [26]), for pattern formation; in particular for branching structures in plants ([27,28] or [29]), lungs ([21]), and other vascular systems ([30,31]). Figure 1 and Figure 2 show numerical simulations for a particular case of the linearization of the Monge–Ampère equation. Growth is induced by the concentration of the solution, and it can be seen that the process gives rise to lateral branches.

## 5. Murray’s Law and Neural Branching

In the previous section, we argued that neuronal branching is compatible with reaction diffusion processes. On the other hand, we deduced the corresponding equations by considering the transport of information along neural networks. The question of whether there is some connection with Murray’s law arises naturally. Recall that Murray’s law refers to a transport network ([32,33]).

In his original paper [32], Murray obtained from optimizing considerations a relationship for the different parameters associated with a branching transport network that was later generalized in [15]. In what follows, we deduce it from scratch for the sake of completeness (we refer the reader to [15,34,35] for further details). The total power required for the flow to overcome the viscous drag is described by:

(27) $$W_{t}=\frac{8\mu Lf^{2}}{\pi r^{4}}+m\pi Lr^{2},$$

where μ is the dynamic viscosity of the fluid, *L* is the vessel length, *f* is the volumetric flow rate, *m* is an all-encompassing metabolic coefficient that includes the chemical cost of keeping the blood constituents fresh and functional and the general cost owing to the weight of the blood and the vessel, and *r* is the vessel radius. For our purposes, we modify Equation (27) as follows:

(28) $$W_{t}=\frac{af^{2}}{r^{2}}+br^{\alpha },$$

where the first term corresponds to the power required for an electrical impulse to propagate along the axon. Notice in particular that the factor of $r^{2}$ in the denominator follows from the fact that electrical resistance is inversely proportional to the area of the section of the conducting material. On the other hand, the second term is proportional to a power, α, of the radius and describes the fact that metabolic cost can vary depending on the type of neurons with which we are dealing. For instance, the degree of myelination of the axon could determine the effective cost associated with information transport. The minimum power is found by differentiating with respect to *r* and equating to zero:

(29) $$\frac{dW_{t}}{dr}=\frac{-2af^{2}}{r^{3}}+\alpha br^{\alpha -1}.$$

With this, the optimal radius:

(30) $$r^{2+\alpha }=\frac{2a}{\alpha b}f^{2},$$

and the optimal relation between volumetric flow rate and vessel radius, such that the power requirement is minimized, is obtained with:

(31) $$f=kr^{1+\frac{\alpha }{2}},$$

where $k=\sqrt{\frac{\alpha b}{2a}}$.

**Figure 3:**
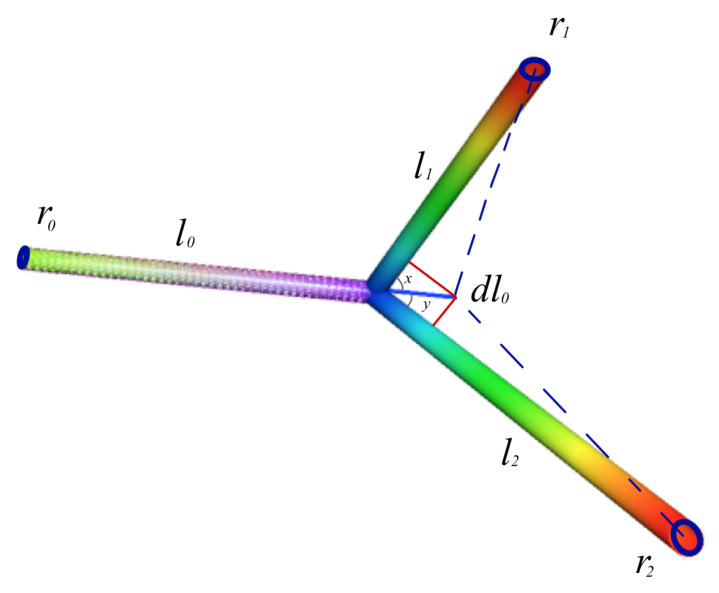
The figure shows schematically the geometric configuration when branching occurs on a plane. Illustration after [33] by the authors.

Using the construction of [33], if the radius of the main branch ($r_{0}$), lateral branches ($r_{1}$, $r_{2}$), and *x* and *y* are the angles between the lateral branches and the main branch, we obtain a generalized version of Murray’s law:

$$f_{0}=f_{1}+f_{2}=kr_{o}^{1+\frac{\alpha }{2}}=k\left(r_{1}^{1+\frac{\alpha }{2}}+r_{2}^{1+\frac{\alpha }{2}}\right);$$

thus, we get the general law:

$$r_{0}^{1+\frac{\alpha }{2}}=r_{1}^{1+\frac{\alpha }{2}}+r_{2}^{1+\frac{\alpha }{2}}.$$

for α∈R. Using these relations, we obtain three general equations associated with the branching angles (see Figure 3) *x*, *y*, and x+y:

(32) $$\cos\left(x\right)=\frac{r_{o}^{4}+r_{1}^{4}-{\left(r_{0}^{1+\frac{\alpha }{2}}-r_{1}^{1+\frac{\alpha }{2}}\right)}^{\frac{8}{2+\alpha }}}{2r_{0}^{2}r_{1}^{2}},$$

(33) $$\cos\left(y\right)=\frac{r_{o}^{4}+r_{2}^{4}-{\left(r_{0}^{1+\frac{\alpha }{2}}-r_{2}^{1+\frac{\alpha }{2}}\right)}^{\frac{8}{2+\alpha }}}{2r_{0}^{2}r_{2}^{2}},$$

(34) $$\cos\left(x+y\right)=\frac{{\left(r_{1}^{1+\frac{\alpha }{2}}+r_{2}^{1+\frac{\alpha }{2}}\right)}^{\frac{8}{2+\alpha }}-r_{1}^{4}-r_{2}^{4}}{2r_{1}^{2}r_{2}^{2}},$$

which correspond to a different generalized Murray’s law for different values of α∈R.

**Observation** **3.** *If α=2, then by *(32)–(34)*, we get cos(x+y)=1 and then x+y=0, which is consistent with the fact that cosx=1, then x=0 and cosy=1, and then, y=0. This case corresponds to no branching. In terms of the cost, this would imply that it is more efficient for the network not to bifurcate.*

*If α=4, then $\cos\left(x\right)=\frac{r_{o}^{4}+r_{1}^{4}-{\left(r_{0}^{3}-r_{1}^{3}\right)}^{\frac{4}{3}}}{2r_{0}^{2}r_{1}^{2}}$, $\cos\left(y\right)=\frac{r_{o}^{4}+r_{2}^{4}-{\left(r_{0}^{3}-r_{2}^{3}\right)}^{\frac{4}{3}}}{2r_{0}^{2}r_{2}^{2}}$, and $\cos\left(x+y\right)=\frac{{\left(r_{1}^{3}+r_{2}^{3}\right)}^{\frac{4}{3}}-r_{1}^{4}-r_{2}^{4}}{2r_{1}^{2}r_{2}^{2}}$, which corresponds to Murray’s original proposal [33], which states that the angle in the bifurcation of an artery should not be less than $75^{\circ }$ ($74.9^{\circ }$ to be more exact). This is consistent with the numerical and experimental results in [36].*

*If α=6, we get cos(x+y)=0 and then $x+y=\frac{\pi }{2}$. On the other hand, $\cos\left(x\right)=r_{1}^{2}/r_{0}^{2}$, then cos(x)>0 since $r_{0},r_{1}\neq 0$; this implies that cos(x)≠0 and $x\neq \frac{\pi }{2}$. Similarly, $y\neq \frac{\pi }{2}$. We obtain that $x+y=\frac{\pi }{2}$ and $x,y\in (0,\frac{\pi }{2})$ for this case. In other words, for α=6, the angle between the bifurcated branches is π/2, but orthogonal branching is ruled out.*

*We conclude then that the relevant values for our purposes are for α∈[2,6]. It would be interesting to contrast these possible scenarios with experimental data for different kinds of nervous tissues. To our knowledge, no systematic experimental study of branching angles has been carried out.*

## 6. Conclusions

We proposed that information flow in some brain processes can be analyzed in the framework of optimal transportation theory. A Monge–Ampère equation was obtained for the optimal transportation plan potential in the one-dimensional case. Extrapolating to higher dimensions, the corresponding linearization around a quadratic distance cost was derived and shown to be consistent with the branching structure of the nervous system. Finally, a generalized version of Murray’s law was derived assuming different cost functions, depending on a parameter related to the metabolic maintenance term. Future work includes a detailed comparison of the methodological proposal with experimental data. In particular, it would be interesting to carry out the program here proposed in a concrete cognitive experiment. Possible concrete experiments to compare with could be found in [2,3,5]. Here, we outline a simple procedure with fMRI data in which a direct connection with optimal transport theory can be tested. Consider the brain activity map for the resting state given by standard fMRI. Once normalized, this map will provide the initial probability density and entropy to be transported. In fact, in [37], another possible methodology for measuring the entropy with fMRI can be found. Later on, the subject is asked to perform a simple motor task, for instance move the right hand. The corresponding density after the task is done can then be registered as before and will provide the final density in the optimal transport problem. Some intermediate densities should be determined as well. This information will provide a transport plan that can be compared with the mathematical solution of the problem. Correspondingly, the branching structures and their bifurcation angles and radii should be compared with experimental results as well. In principle, Murray’s law should be consistent with the Monge–Ampère equation and its linearization, and it should be possible to derive it from them. A precise relationship between the maximum entropy principle and optimal entropy transport should be clarified.

## Appendix A. Relevant Theorems and Some Proofs

**Theorem** **A1** (The continuity and support theorem)**.** *Let μ be a probability measure on R with cumulative distribution function F. The following properties are equivalent:*

a. *The function F is strictly increasing in the interval:*
  {x∈R|0<F(x)<1}.
b. The inverse function $F^{-1}$ is continuous.
c. The inverse measure $\mu^{-1}$ is non-atomic.
d. *The support of μ, given by:*
  $$supp\left(\mu \right)=\bar{\left\{A\in \Sigma \;|\;\mu (A)>0\right\}}$$
  *with *Σ* a σ-algebra defined on R, is a closed interval in the real line, finite or not.*

**Proof.** The proof of this result can be found in [38], Appendix A. □

**Proposition** **A1.**
*Let h *∈*$C^{1}\left(R\right)$ be a non-negative, strictly convex function. Let μ and ν be Borel probability measures on R given by *(11)* and *(12)*, respectively. Suppose that:*

(A1) $$\int_{\Omega }\int_{\Omega }h\left(x-y\right)d\mu \left(x\right)d\nu \left(y\right)<\infty .$$

*for Ω∈R. If μ has no atom and F and G represent the respective cumulative distribution functions of μ and ν, respectively, then:*

$$T=G^{-1}\circ F$$

*solves Problem *(14)* for the cost:*

c(x,y)=h(x−y).

*If π is the induced entropy transform plan, that is:*

π=(Id,T)#μ,

*defined as $T\#\mu \left(E\right)=\mu (T^{-1}\left(E\right))$ for E∈Ω, then π is optimal for Problem *(15)*.*

**Proof.** 

1. T is well defined:
  The only problem we might have with the definition of $T=G^{-1}\circ F$ could be when F(x)=0. However, if F(x)=0, then:
  $$\begin{array}{cc} T(x) & =G^{-1}\left(F\left(x\right)\right)\\ & =G^{-1}\left(0\right)\\ & =\min\left\{y\in [-\infty ,\infty ]\;|\;0\leq G(y)\right\}\\ & =-\infty , \end{array}$$
  but if for some a∈R, we have F(a)=0, then μ((−∞,a])=0, which means that a=−∞ and *T* is well defined, as desired.
2. ν=T#μ:
  Let *F* and *G* be defined as in observation (1). Then, $T=G^{-1}\circ F$ is non-decreasing, since *F* and *G* are non-decreasing. Then:
  $$\begin{array}{cc} T^{-1}\left(\left(-\infty ,y\right]\right) & =\left\{x\in [-\infty ,\infty ]\;|\;T(x)=y\right\}\\ & =\left\{x\in \left[-\infty ,\infty \right]\;|\;G^{-1}\left(F\left(x\right)\right)=y\right\}\\ & =\left\{x\in [-\infty ,\infty ]\;|\;F(x))=G(y)\right\}, \end{array}$$
  Since *T* is non-decreasing, $T^{-1}\left(\left(-\infty ,y\right]\right)$ is an interval.
  Claim 1. Since μ has no atom, *F* is increasing and continuous, and then, $T^{-1}\left(\left(-\infty ,y\right]\right)$ is a closed interval.
  If μ has no atom, let A∈Ω and $\alpha \in R^{+}$ such that μ(A)=α. Then, given $\beta \in R^{+}$ such that 0<β≤α, there exits B⊂A such that μ(B)=β. Then, ∀γ∈R such that 0<γ≤α, there exists $x_{\gamma }\in A$ such that:
  $$\mu (\left(-\infty ,x_{\gamma }\right])=\gamma ,$$
  then $0<F\left(x_{\gamma }\right)<F\left(x_{\alpha }\right)\leq 1$ for all γ,α such that $x_{\gamma }<x_{\alpha }$, and so, *F* is increasing and continuous; Theorem A1 implies that $F^{-1}$ is continuous, and then:
  {x∈R|0≤F(x)≤1}=supp(μ),
  is closed. In particular, {x∈R|0≤F(x)≤G(y)} is closed. Then, we conclude that $T^{-1}\left(\left(-\infty ,y\right]\right)$ is a closed interval, as desired.
  We have proven Claim 1.
  Now, if x=sup{x∈R|F(x)≤G(y)}, then F(x)=G(y), and we have:
  $$\begin{array}{cc} \mu \left(T^{-1}\left(\left(-\infty ,y\right]\right)\right) & =\mu \left((-\infty ,x]\right)\\ & =F(x)\\ & =G(y)\\ & =\nu \left((-\infty ,y]\right), \end{array}$$
  and then, ν=T#μ, as desired.
3. $T=G^{-1}\circ F$ is optimal:
  Observe that (μ,ν) given by (11) and (12) satisfy:
  $$\begin{array}{c} \int_{Y=\Omega }d\pi \left(x,y\right)=d\mu \left(x\right) \end{array}\;\mathrm{and}\;\begin{array}{c} \int_{X=\Omega }d\pi \left(x,y\right)=d\nu \left(y\right) \end{array}$$
  where:
  $$d\pi \left(x,y\right)=\frac{1}{K}|\rho \left(x,y\right)\ln(\rho \left(x,y\right)|dxdy,$$
  and:
  $$d\mu \left(x\right)=\frac{1}{K}|\rho \left(x\right)\ln\left(\rho \left(x\right)\right)|dx,$$
  then $\pi \left(x,y\right)\in \hat{\Pi }\left(\mu ,\nu \right)$ as given by (13).
  Now, observe that:
  $$\begin{array}{cc} \int_{\Omega }h^{\prime }\left(x-T\left(x\right)\right)\frac{1}{K}|\rho \left(x\right)\ln\left(\rho \left(x\right)\right)|dx & =\int_{\Omega }h^{\prime }\left(x-T\left(x\right)\right)d\mu ; \end{array}$$
  since *T* and $h^{\prime }$ are non-decreasing,
  $$h^{\prime }\left(u-T\left(u\right)\right)\leq h^{\prime }\left(u-T\left(x\right)\right)$$
  for u≥x. Then:
  $$\begin{array}{cc} \int_{x}^{y}h^{\prime }\left(u-T\left(u\right)\right)du & \leq \int_{x}^{y}h^{\prime }\left(u-T\left(x\right)\right)du\\ & \leq h(y-T(x))-h(x-T(x)). \end{array}$$
  On the other hand,
  $$h^{\prime }\left(u-T\left(u\right)\right)\geq h^{\prime }\left(u-T\left(x\right)\right)$$
  for u≤x, and then:
  $$\begin{array}{cc} \int_{x}^{y}h^{\prime }\left(u-T\left(u\right)\right)du & =-\int_{y}^{x}h^{\prime }\left(u-T\left(u\right)\right)du\\ & \leq -\int_{y}^{x}h\left(u-T\left(x\right)\right)du;\\ & =-[h(x-T(x))-h(y-T(x))]\\ & =h(y-T(x))-h(x-T(x)). \end{array}$$
  As a consequence, in any case, we have:
  (A2) $$\begin{array}{c} \int_{x}^{y}[h\left(u-T\left(u\right)\right)du\leq h\left(y-T\left(x\right)\right)-h\left(x-T\left(x\right)\right). \end{array}$$
  Set:
  $$\psi \left(y\right)=\int_{0}^{y}h^{\prime }\left(u-T\left(u\right)\right)du,$$
  then (A2) becomes:
  ψ(y)−ψ(x)≤h(y−T(x))−h(x−T(x)),
  which implies:
  $$\begin{array}{cc} \psi^{c}\left(T\left(x\right)\right) & =\underset{y}{\inf}\left\{h(y-T(x))-\psi (y)\right\}\\ & =h(x-T(x))-\psi (x); \end{array}$$
  as a consequence, ψ is *c*-concave, according to Definition (1).
  Hypothesis A1 implies the existence of $x_{0}$, $y_{0}\in \Omega$ such that:
  (A3) $$\begin{array}{c} \int_{\Omega }h\left(x-y_{0}\right)d\mu \left(x\right)<\infty \end{array}\;\mathrm{and}\;\begin{array}{c} \int_{\Omega }h\left(x_{0}-y\right)d\nu \left(y\right)<\infty . \end{array}$$
  Claim 2: $\psi \in L^{1}\left(\mu \right)$ and $\psi^{c}\in L^{1}\left(\nu \right)$: Observe that:
  (A4) $$\begin{array}{cc} h\left(x-y_{0}\right)-\psi^{c}\left(y_{0}\right)\; & =h\left(x-y_{0}\right)-\underset{y}{\inf}\left\{h\left(y-y_{0}\right)-\psi \left(y\right)\right\}\\ \; & \geq h\left(x-y_{0}\right)-\left(h\left(x-y_{0}\right)-\psi \left(x\right)\right)\\ \; & =\psi (x), \end{array}$$
  and:
  $$\begin{array}{cc} h\left(x_{0}-T\left(x\right)\right)-\psi \left(x_{0}\right) & \geq \underset{y}{\inf}\left\{h(y-T(x))-\psi (y)\right\}\\ & =h(x-T(x))-\psi (x)\\ & =\psi^{c}\left(T\left(x\right)\right); \end{array}$$
  hence:
  (A5) $$\psi \left(x\right)\geq -\psi^{c}\left(T\left(x\right)\right)\geq -h\left(x_{0}-T\left(x\right)\right)+\psi \left(x_{0}\right),$$
  then, by (A4) and (A5),
  (A6) $$\begin{array}{c} h\left(x-y_{0}\right)-\psi^{c}\left(y_{0}\right)\geq \psi \left(x\right)\geq -h\left(x_{0}-T\left(x\right)\right)+\psi \left(x_{0}\right); \end{array}$$
  now, by Hypothesis A3 and since ν=T#μ:
  (A7) $$\int_{\Omega }h\left(x_{0}-T\left(x\right)\right)d\mu =\int_{\Omega }h\left(x_{0}-y\right)d\nu <\infty ;$$
  therefore, integrating (A6) with respect to μ, we get:
  $$\int_{\Omega }\left[h\left(x-y_{0}\right)-\psi^{c}\left(y_{0}\right)\right]d\mu \left(x\right)\geq \int_{\Omega }\psi \left(x\right)d\mu \left(x\right)\geq \int_{\Omega }\left[-h\left(x_{0}-T\left(x\right)\right)+\psi \left(x_{0}\right)\right]d\mu \left(x\right);$$
  as a consequence:
  $$\int_{\Omega }\left|\psi \left(x\right)\right|d\mu \left(x\right)<\infty ,$$
  and we can conclude that $\psi \in L^{1}\left(\mu \right)$.
  Similarly:
  $$\begin{array}{cc} h\left(x_{0}-T\left(x\right)\right)-\psi \left(x_{0}\right)\geq \psi^{c}\left(T\left(x\right)\right) & \geq -\psi \left(x\right)\geq -h\left(x-y_{0}\right)+\psi^{c}\left(y_{0}\right) \end{array}$$
  by (A4) and (A5); using (A3) and (A7), if we integrate with respect to μ,
  $$\int_{\Omega }\left[h\left(x_{0}-T\left(x\right)\right)-\psi \left(x_{0}\right)\right]d\mu \left(x\right)\geq \int_{\Omega }\psi^{c}\left(T\left(x\right)\right)d\mu \left(x\right)\geq \int_{\Omega }\left[-h\left(x-y_{0}\right)+\psi^{c}\left(y_{0}\right)\right]d\mu \left(x\right);$$
  therefore:
  $$\int_{\Omega }|\psi^{c}\left(T\left(x\right)\right)|d\mu \left(x\right)=\int_{\Omega }\left|\psi^{c}\left(y\right)\right|d\nu \left(y\right)<\infty$$
  Hence, $\psi^{c}\in L^{1}\left(\nu \right)$. We have proven Claim 2.
  Integrating $\phi \left(x\right)+\phi^{c}\left(T\left(x\right)\right)=h\left(x-T\left(x\right)\right)=c\left(x,y\right)$ with respect to μ, we get:
  $$\int_{\Omega }\psi \left(x\right)d\mu \left(x\right)+\int_{\Omega }\psi^{c}\left(T\left(x\right)\right)d\mu \left(x\right)=\int_{\Omega }h\left(x-T\left(x\right)\right)d\mu \left(x\right),$$
  and then:
  $$\int_{\Omega }\psi \left(x\right)d\mu \left(x\right)+\int_{\Omega }\psi^{c}\left(y\right)d\nu \left(y\right)=\int_{\Omega }c\left(x,T\left(x\right)\right)d\mu \left(x\right).$$
  Finally, observe that $\psi \left(x\right)+\psi^{c}\left(y\right)\leq c\left(x,y\right)$ for every (x,y)∈Ω×Ω; if $\pi_{1}\in \hat{\Pi }\left(\mu ,\nu \right)$ is another entropy transport plan, the associated total entropy transport cost c(x,y) is greater, by the definition of ψ and $\psi^{c}$; then, the equality holds only for the optimal entropy transport plan π and $T=G^{-1}\circ F$; hence, it solves Problem (14), and π=(Id,T)#μ solves Problem (15).
  We have proven the proposition.
   □

**Proposition** **A2.**
*Let μ and ν be the Borel probability measures on Ω∈R given by *(11)* and *(12)*, respectively. If the cost function:*

c:R×R⟶[0,∞)

*is lower semi-continuous and:*

$$\int_{\Omega }\int_{\Omega }c\left(x,y\right)d\mu \left(x\right)d\nu \left(y\right)<\infty ,$$

*then there is a Borel map ψ:R⟶R that is c-concave and optimal for *(3)*. Moreover, the resulting maximum is equal to the minimum of Problem *(15)*; i.e.,*

$$\min_{\pi \in \hat{\Pi }\left(\mu ,\nu \right)}\int_{R\times R}c\left(x,y\right)d\pi \left(x,y\right)=\max_{\psi \in L^{1}\left(\mu \right)}\left\{\int_{\Omega }\psi \left(x\right)d\mu \left(x\right)+\int_{\Omega }\psi^{c}\left(y\right)d\nu \left(y\right)\right\}$$

*or:*

(A8) $$\min_{\pi \in \hat{\Pi }\left(\mu ,\nu \right)}I\left[\pi \right]=\max_{(\psi ,\psi^{c})\in \Phi }J\left(\psi ,\psi^{c}\right),$$

*where,*

(A9) $$\Phi =\left\{\left(\phi ,\psi \right)\in L^{1}\left(\mu \right)\times L^{1}\left(\nu \right)\;|\;\phi \left(x\right)+\psi \left(y\right)\leq c\left(x,y\right)\;\mathrm{for}\;\mu -a.e.\;x\in \Omega \;\mathrm{and}\;\nu -a.e.\;y\in \Omega \right\},$$

*and if $\pi \in \hat{\Pi }\left(\mu ,\nu \right)$ given by *(13)* is optimal, then $\psi \left(x\right)+\psi^{c}\left(y\right)=c\left(x,y\right)$ almost everywhere for π.*

**Proof.** The existence of a maximizing pair and Relation (A8) have been proven in Proposition A1. Now, choosing an optimal $\pi \in \hat{\Pi }\left(\mu ,\nu \right)$, we have:

$$\begin{array}{ccc} \int_{\Omega \times \Omega }\left[c\left(x,y\right)-\psi \left(x\right)-\psi^{c}\left(y\right)\right]d\pi \left(x,y\right)\; & =\int_{\Omega \times \Omega }c\left(x,y\right)d\pi \left(x,y\right)\; & -\int_{\Omega }\psi \left(x\right)d\mu \left(x\right)\\ \; & \;-\int_{\Omega }\psi^{c}\left(y\right)d\nu \left(y\right)\\ \; & =0, \end{array}$$

and since $c\left(x,y\right)-\psi \left(x\right)-\psi^{c}\left(y\right)\geq 0$ in Ω×Ω, it must vanish for π-a.e. (x,y)∈Ω×Ω. The proof is completed. □

**Theorem** **A2** (Brenier)**.** *Let μ and ν be the Borel probability measures on Ω∈R given by *(11)* and *(12)*, respectively, and with second-order moments; that is, such that:*

(A10) $$\begin{array}{c} \int_{\Omega }{\left|x\right|}^{2}d\mu \left(x\right)<\infty \end{array}\;\mathrm{and}\;\begin{array}{c} \int_{\Omega }{\left|y\right|}^{2}d\nu \left(y\right)<\infty \end{array}$$

*Then, if μ is absolutely continuous on *Ω*, there exists a unique T:R⟶R such that ν=T#μ and:*

$$\int_{\Omega }{\left|x-T\left(x\right)\right|}^{2}d\mu \left(x\right)=\min_{\gamma \in \hat{\Pi }\left(\mu ,\nu \right)}\int_{\Omega }{\left|x-y\right|}^{2}d\gamma \left(x,y\right),$$

*with $\hat{\Pi }\left(\mu ,\nu \right)$ given by *(13)*. Moreover, there is only one optimal transport plan γ, which is necessarily (Id,T)#μ, and T is the gradient of a convex function φ, which is therefore unique up to an additive constant. There is also a unique (up to an additive constant) Kantorovich potential, ψ, which is locally Lipschitz and linked to φ through the relation:*

$$\varphi \left(x\right)=\frac{1}{2}{\left|x\right|}^{2}-\psi \left(x\right).$$

**Proof.** Claim 1: π=(Id,∇ϕ)#μ, and it is optimal: Set $c\left(x,y\right)=\frac{{\left|x-y\right|}^{2}}{2}$. Proposition A1 guarantees the existence of the pair $(\psi ,\psi^{c})\in \Phi$ as given by (A9) such that $\psi \left(x\right)+\psi^{c}\left(y\right)\leq \frac{{\left|x-y\right|}^{2}}{2}$ for all (x,y)∈Ω×Ω; then:

$$\psi \left(x\right)+\psi^{c}\left(y\right)\leq \frac{x^{2}-2xy+y^{2}}{2}$$

and then:

$$xy\leq \left[\frac{x^{2}}{2}-\psi \left(x\right)\right]+\left[\frac{y^{2}}{2}-\psi^{c}\left(y\right)\right].$$

Set:

(A11) $$\begin{array}{c} \varphi \left(x\right)=\left[\frac{x^{2}}{2}-\psi \left(x\right)\right] \end{array}\;\mathrm{and}\;\begin{array}{c} \varphi^{c}\left(y\right)=\left[\frac{y^{2}}{2}-\psi^{c}\left(y\right)\right] \end{array}.$$

Hypothesis A10 means:

$$M=\int_{\Omega }\frac{{\left|x\right|}^{2}}{2}d\mu \left(x\right)+\int_{\Omega }\frac{{\left|y\right|}^{2}}{2}d\nu \left(y\right)<\infty ,$$

then:

$$I\left[\pi \right]=\int_{\Omega \times \Omega }\frac{{\left|x-y\right|}^{2}}{2}d\pi \left(x,y\right)\leq \int_{\Omega \times \Omega }\left[{\left|x\right|}^{2}+{\left|y\right|}^{2}\right]d\pi \left(x,y\right)=2M;$$

hence, I[π] is always finite in $\hat{\Pi }\left(\mu ,\nu \right)$ given by (13). As a consequence,

$$\begin{array}{cc} \underset{\hat{\Pi }\left(\mu ,\nu \right)}{\inf}I\left[\pi \right] & =\frac{1}{2}\left[\int_{\Omega }{\left|x\right|}^{2}d\mu \left(x\right)+\int_{\Omega }{\left|y\right|}^{2}d\nu \left(y\right)\right]\\ & \;-\sup\left\{\int_{\Omega \times \Omega }xy\;d\pi \left(x,y\right)\;|\;\pi \in \hat{\Pi }\left(\mu ,\nu \right)\right\}\\ & =M-\underset{\hat{\Phi }}{\inf}\left\{\int_{\Omega }\varphi \left(x\right)\;d\mu \left(x\right)+\int_{\Omega }\varphi^{c}\left(y\right)\;d\nu \left(y\right)\right\}\\ & =\underset{\Phi }{\sup}\left\{\int_{\Omega }\varphi \left(x\right)\;d\mu \left(x\right)+\int_{\Omega }\varphi^{c}\left(y\right)\;d\nu \left(y\right)\right\}\\ & =\underset{\Phi }{\sup}J\left(\varphi ,\varphi^{c}\right), \end{array}$$

where $J\left(\varphi ,\varphi^{c}\right)=\int_{\Omega }\varphi \left(x\right)\;d\mu \left(x\right)+\int_{\Omega }\varphi^{c}\left(y\right)\;d\nu \left(y\right)$, Φ given by (18), and:

(A12) $$\begin{array}{c} \hat{\Phi }=\{\left(\varphi ,\psi \right)\in L^{1}\left(\mu \right)\times L^{1}\left(\nu \right)\;\mathrm{with}\;\mathrm{values}\;\mathrm{in}\;R\cup \left\{\infty \right\}\;|\;xy\leq \varphi \left(x\right)+\psi \left(y\right)\;\mathrm{for}\;\mu -a.e.\;x\in \Omega \\ \mathrm{and}\;\nu -a.e.\;y\in \Omega \}. \end{array}$$

Then, the Kantorovich duality principle (4) becomes:

(A13) $$\sup\left\{\int_{\Omega \times \Omega }xy\;d\pi \left(x,y\right)\;|\;\pi \in \hat{\Pi }\left(\mu ,\nu \right)\right\}=\underset{\hat{\Phi }}{\inf}J\left(\varphi ,\varphi^{c}\right).$$

Now, $xy\leq \varphi \left(x\right)+\varphi^{c}\left(y\right)$ since $\left(\varphi ,\varphi^{c}\right)\in \hat{\Phi }$; hence:

$$\varphi^{c}\geq \underset{x}{\sup}\left\{xy-\varphi (x)\right\}\equiv \varphi^{*}\left(y\right).$$

$\varphi^{*}\left(y\right)$ is called the Legendre transform of φ(x) and satisfies:

$$\varphi \left(x\right)+\varphi^{*}\left(y\right)\geq xy.$$

It can be proven that $\underset{\hat{\Phi }}{\inf}J=J\left(\varphi ,\varphi^{*}\right)$ (see [16]; Theorem 2.9); now, Proposition A1 guarantees the existence of an optimal transport plan π. As a consequence, Relation (A13) implies:

$$\begin{array}{cc} \int_{\Omega }\varphi \left(x\right)\;d\mu \left(x\right)+\int_{\Omega }\varphi^{*}\left(y\right)\;d\nu \left(y\right) & =\int_{\Omega \times \Omega }xy\;d\pi \left(x,y\right)\\ & =\int_{\Omega \times \Omega }\left[\varphi \left(x\right)+\varphi^{c}\left(y\right)\right]\;d\pi \left(x,y\right), \end{array}$$

and then:

$$\int_{\Omega \times \Omega }\left[\varphi \left(x\right)+\varphi^{c}\left(y\right)-xy\right]\;d\pi \left(x,y\right)=0,$$

for all (x,y)∈Ω×Ω. However, $\varphi \left(x\right)+\varphi^{c}\left(y\right)-xy\geq 0$ by the definition of the Legendre transform. Then, for π-a.e. (x,y)∈Ω×Ω, we have:

(A14) $$\begin{array}{cc} \varphi \left(x\right)+\varphi^{*}\left(y\right)-xy=0 & ⟺xy\geq \varphi \left(x\right)+\varphi^{*}\left(y\right)\\ \; & ⟺xy\geq \varphi \left(x\right)+\left(zy-\varphi (z)\right),\;\forall z\in R\\ \; & ⟺\varphi (z)\geq \varphi (x)+y(z-x)\\ \; & ⟺y\in \partial \varphi (x) \end{array}$$

(by symmetry, we can also conclude that $x\in \;\partial \varphi^{*}\left(y\right)$). Now, since φ is convex, it can be proven that it is locally Lipschitz and continuous; Rademacher’s theorem (see [39]; Section 3.1.2) implies then that φ is differentiable μ-a.e. on R. As a consequence:

(A15) $$\partial \varphi \left(x\right)=\left\{\nabla \varphi (x)\right\}.$$

One can prove that y=∇φ(x) (see [16]). Therefore, π=(Id,T)#μ=(Id,∇φ)#μ, and it is optimal. We have proven Claim 1. Claim 2: π=(Id,∇φ)#μ is the only transport plan: Let $\hat{\varphi }$ be another convex function such that $\nu =\nabla \hat{\varphi }\#\mu$. We want to prove $\nabla \varphi \left(x\right)=\nabla \hat{\varphi }\left(x\right)$ μ-a.e. x∈Ω. By Claim 1, $(Id,\nabla \hat{\varphi })\#\mu$ is an optimal transport plan, and the pair $\left(\hat{\varphi },{\hat{\varphi }}^{*}\right)$ is optimal for the dual problem, just like $\left(\varphi ,\varphi^{*}\right)$. Therefore:

$$\int_{\Omega }\hat{\varphi }\left(x\right)d\mu \left(x\right)+\int_{\Omega }{\hat{\varphi }}^{*}\left(y\right)d\nu \left(y\right)=\int_{\Omega }\varphi \left(x\right)d\mu \left(x\right)+\int_{\Omega }\varphi^{*}\left(y\right)d\nu \left(y\right).$$

Let π be the optimal transport plan associated with φ. Then:

$$\int_{\Omega \times \Omega }\left[\hat{\varphi }\left(x\right)+{\hat{\varphi }}^{*}\left(y\right)\right]d\pi \left(x,y\right)=\int_{\Omega \times \Omega }\left[\varphi \left(x\right)+\varphi^{*}\left(y\right)\right]d\pi \left(x,y\right)=\int_{\Omega \times \Omega }xy\;d\pi \left(x,y\right);$$

hence:

$$\int_{\Omega }\left[\hat{\varphi }\left(x\right)+{\hat{\varphi }}^{*}\left(\nabla \varphi \left(x\right)\right)\right]d\mu \left(x\right)=\int_{\Omega }x\nabla \varphi \left(x\right)d\mu \left(x\right),$$

and:

$$\int_{\Omega }\left[\hat{\varphi }\left(x\right)+{\hat{\varphi }}^{*}\left(\nabla \varphi \left(x\right)\right)-x\nabla \varphi \left(x\right)\right]d\mu \left(x\right)=0;$$

since $\hat{\varphi }\left(x\right)+{\hat{\varphi }}^{*}\left(\nabla \varphi \left(x\right)\right)-x\nabla \varphi \left(x\right)\geq 0$, we conclude:

$$\hat{\varphi }\left(x\right)+{\hat{\varphi }}^{*}\left(\nabla \varphi \left(x\right)\right)-x\nabla \varphi \left(x\right)=0,$$

μ-a.e. on Ω. Hence, by (A14):

$$\nabla \varphi \left(x\right)\in \partial \hat{\varphi }\left(x\right),\;\mu -a.e.\;x\in \Omega .$$

Now, since $\hat{\varphi }$ is differentiable μ-a.e. on Ω:

$$\nabla \varphi \left(x\right)=\nabla \hat{\varphi }\left(x\right).$$

Claim 2 has been proven. Finally, we have proven not only the uniqueness of Problem (15) (analogous to the Monge–Kantorovich problem), but also the uniqueness of the gradient of a convex function ∇ϕ such that ∇φ#μ=ν and:

$$\min_{\gamma \in \hat{\Pi }\left(\mu ,\nu \right)}\int_{\Omega \times \Omega }{\left|x-y\right|}^{2}d\gamma \left(x,y\right)=\int_{\Omega }{\left|x-\nabla \varphi \left(x\right)\right|}^{2}d\mu \left(x\right)=\int_{\Omega }{\left|x-T\left(x\right)\right|}^{2}d\mu \left(x\right).$$

We have proven the theorem. □

## Appendix B. Linearization of the Monge–Ampère Equation

Assume that φ is very close to $\frac{{\left|x\right|}^{2}}{2}$, so |ρ(x)ln[ρ(x)]| is very close to $\left|\tilde{\rho }\left(\nabla \varphi \left(x\right)\right)\ln\left[\tilde{\rho }\left(\nabla \varphi \left(x\right)\right)\right]\right|$. In that case, following [16,22,23], make:

$$\varphi \left(x\right)\equiv \varphi_{\varepsilon }\left(x\right)=\frac{{\left|x\right|}^{2}}{2}+\varepsilon \eta +O\left(\varepsilon^{2}\right),$$

and:

$$|\tilde{\rho }\left(\nabla \varphi \left(x\right)\right)\ln\left[\tilde{\rho }\left(\nabla \varphi \left(x\right)\right)\right]|\equiv |\tilde{\rho }\left(\nabla \varphi \left(x\right)\right)\ln\left[\tilde{\rho }\left(\nabla \varphi \left(x\right)\right)\right]{|}_{\varepsilon }=\left(1+\varepsilon h+O\left(\varepsilon^{2}\right)\right)|\rho \left(x\right)\ln\left[\rho \left(x\right)\right]|.$$

with $\eta ,h\in L^{1}\left(\mu \right)$. Then, by the Neumann series:

(A16) $$\begin{array}{cc} \frac{\left|\rho (x)\ln[\rho (x)]\right|}{\left|\tilde{\rho }\left(\nabla \varphi \left(x\right)\right)\ln\left[\tilde{\rho }\left(\nabla \varphi \left(x\right)\right)\right]\right|}\; & =\frac{\left|\rho (x)\ln[\rho (x)]\right|}{\left(1+\varepsilon h+O\left(\varepsilon^{2}\right)\right)|\rho \left(\nabla \varphi \left(x\right)\right)\ln\left[\rho \nabla \varphi \left(x\right)\right)]|}\\ \; & \\ \; & =\frac{\left(1-\varepsilon h+O\left(\varepsilon^{2}\right)\right)|\rho \left(x\right)\ln\left[\rho \left(x\right)\right]|}{\left|\rho (\nabla \varphi (x))\ln[\rho \nabla \varphi (x))]\right|}. \end{array}$$

On the other hand, it is easy to see that:

$$\begin{array}{cc} \det D^{2}\varphi \left(x\right) & =\det D^{2}\left(\frac{{\left|x\right|}^{2}}{2}+\varepsilon \eta +O\left(\varepsilon^{2}\right)\right)\\ \; & =\det D^{2}\left(\frac{{\left|x\right|}^{2}}{2}\right)+\varepsilon \mathrm{trace}\left(HD^{2}\eta \right)+O\left(\varepsilon^{2}\right). \end{array}$$

**Definition** **A1.**
*The term $\mathrm{trace}\left({\mathrm{HD}}^{2}\eta \right)$ is called the linearization of the Monge–Ampère equation, where $H={\left(H\right)}_{ij}$ is the matrix of co-factors of $D^{2}\left(\frac{{\left|x\right|}^{2}}{2}\right)=I$.*

Then:

(A17) $$\det D^{2}\left(\varphi (x)\right)=1+\varepsilon \mathrm{trace}\left(D^{2}\eta \right)+O\left(\varepsilon^{2}\right).$$

Substituting (A16) and (A17) into the Monge–Ampère Equation (22) and omitting order superior terms, we get:

(A18) $$\begin{array}{cc} (1+\varepsilon \Delta \eta )|\rho (\nabla \varphi (x))\ln[\rho \nabla \varphi (x))]|\; & =(1-\varepsilon h)|\rho (x)\ln[\rho (x)]|, \end{array}$$

and:

$$\begin{array}{cc} |\rho (\nabla \varphi (x))\ln[\rho \nabla \varphi (x))]|\; & =|\rho \ln\rho |(\nabla \varphi (x))\\ \; & =|\rho \ln\rho |\nabla \left(\frac{{\left|x\right|}^{2}}{2}+\varepsilon \eta \right)\\ \; & =|\rho \ln\rho |\left(x+\varepsilon \nabla \eta \right)\\ \; & =|\rho \left(x\right)\ln\rho (x)|+\varepsilon \nabla \eta \cdot \nabla \left(|\rho (x)\ln\rho (x)|\right), \end{array}$$

by using the order one Taylor’s series. as a consequence, (A18) becomes:

$$\begin{array}{cc} \left(1+\Delta \eta \right)\left[|\rho \left(x\right)\ln\rho (x)|+\varepsilon \nabla \eta \cdot \nabla \left(|\rho (x)\ln\rho (x)|\right)\right] & =(1-\varepsilon h)|\rho (x)\ln\rho (x)|, \end{array}$$

then:

$$\begin{array}{cc} \left(1-\varepsilon h\right) & =\left(1+\Delta \eta \right)\left[1+\varepsilon \nabla \eta \cdot \left(\frac{\nabla |\rho (x)\ln\rho (x)|}{\left|\rho (x)\ln\rho (x)\right|}\right)\right]\\ \; & =\left(1+\Delta \eta \right)\left[1+\varepsilon \nabla \eta \cdot \nabla \log|\rho (x)\ln\rho (x)|\right], \end{array}$$

and then:

$$\left(1-\varepsilon h\right)=1+\varepsilon \nabla \eta \cdot \nabla \log|\rho \left(x\right)\ln\rho (x)|+\varepsilon \Delta \eta +\varepsilon^{2}\Delta \eta \left[\nabla \eta \cdot \nabla \log|\rho (x)\ln\rho (x)|\right];$$

omitting again order two terms, we get:

−h=∇η·∇log|ρ(x)lnρ(x)|+Δη,

or simply:

(A19) Lη=h,

with:

$$L=\left(-\Delta +\nabla \left(-\log|\rho (x)\ln\rho (x)|\right)\cdot \nabla \right).$$

We have proven that the Laplace equation can be seen as the linearized version of the Monge–Ampère equation for our proposal.
